# Association of Variants at *BCL11A* and *HBS1L-MYB* with Hemoglobin F and Hospitalization Rates among Sickle Cell Patients in Cameroon

**DOI:** 10.1371/journal.pone.0092506

**Published:** 2014-03-25

**Authors:** Ambroise Wonkam, Valentina J. Ngo Bitoungui, Anna A. Vorster, Raj Ramesar, Richard S. Cooper, Bamidele Tayo, Guillaume Lettre, Jeanne Ngogang

**Affiliations:** 1 Division of Human Genetics, Institute of Infectious Disease and Molecular Medicine, Faculty of Health Sciences, University of Cape Town, Cape Town, Republic of South Africa; 2 Department of Microbiology, Parasitology and Haematology, Faculty of Medicine and Biomedical Sciences, University of Yaoundé 1, Yaoundé, Cameroon; 3 UCT/MRC Human Genetics Research Unit, Division of Human Genetics, Institute of Infectious Disease and Molecular Medicine, Faculty of Health Sciences, University of Cape Town, Cape Town, Republic of South Africa; 4 Department of Public Health Sciences, Loyola University Chicago, Stritch School of Medicine, Maywood, Illinois, United States of America; 5 Montreal Heart Institute and Université de Montréal, Montreal, Quebec, Canada; Instituto de Higiene e Medicina Tropical, Portugal

## Abstract

**Background:**

Genetic variation at loci influencing adult levels of HbF have been shown to modify the clinical course of sickle cell disease (SCD). Data on this important aspect of SCD have not yet been reported from West Africa. We investigated the relationship between HbF levels and the relevant genetic loci in 610 patients with SCD (98% HbSS homozygotes) from Cameroon, and compared the results to a well-characterized African-American cohort.

**Methods and Findings:**

Socio-demographic and clinical features were collected and medical records reviewed. Only patients >5 years old, who had not received a blood transfusion or treatment with hydroxyurea were included. Hemoglobin electrophoresis and a full blood count were conducted upon arrival at the hospital. RFLP-PCR was used to describe the *HBB* gene haplotypes. SNaPshot PCR, Capillary electrophoresis and cycle sequencing were used for the genotyping of 10 selected SNPs. Genetic analysis was performed with PLINK software and statistical models in the statistical package R. Allele frequencies of relevant variants at *BCL11A* were similar to those detected in African Americans; although the relationships with Hb F were significant (p <.001), they explained substantially less of the variance in HbF than was observed among African Americans (∼ 2% vs 10%). SNPs in *HBS1L-MYB* region (*HMIP*) likewise had a significant impact on HbF, however, we did not find an association between HbF and the variations in *HBB* cluster and *OR51B5/6* locus on chromosome 11p, due in part to the virtual absence of the Senegal and Indian Arab haplotypes. We also found evidence that selected SNPs in *HBS1L-MYB* region (*HMIP*) and *BCL11A* affect both other hematological indices and rates of hospitalization.

**Conclusions:**

This study has confirmed the associations of SNPs in *BCL11A* and *HBS1L-MYB* and fetal haemoglobin in Cameroonian SCA patients; hematological indices and hospitalization rates were also associated with specific allelic variants.

## Introduction

Sickle cell disease (SCD) affects the structure of erythrocytes by altering the normal biconcave shape to a crescent. During this process the hemoglobin S (HbS) mutation leads to polymerization and precipitation of hemoglobin during deoxygenation or dehydration resulting in sickling, abnormal adhesion of leukocytes and platelets, inducing inflammation, hypercoagulation, hemolysis and hypoxia, in addition to microvascular obstruction, and ultimately organ damage [1]. SCD is prevalent among indigenous populations in tropical regions of Africa and Asia; 305,800 births with SCD are estimated to occur annually, nearly two-third of which take place in Africa. Sickle Cell Anaemia (SCA; the homozygous HbSS state) is by far the most prevalent and severe form of SCD [2]. Only limited early detection and treatment initiatives have been implemented in Africa and as consequence death rates are high before the age of 5 years in this region [3].

Cameroon has a population about 20 million inhabitants and a growth rates of 3% per annum; the carrier frequency of SCA ranges from 8 to 34% [4]. Cameroon has developed a national control program for SCA, although at present it remains a policy document without implementation; provision of neonatal screening and development of specialized centers for lifelong medical care and surveillance have yet to become part of the health system [5]. Universal medical insurance coverage does not exist, and care of SCA patients is therefore dependent on financial support and care-giving by family members. Poverty in Cameroon affects more than 50% of the rural population and up to 30% of the urban population [6], which in turn means that the financial burden of medical care for SCA can often not be met [5] and patients frequently suffer exceptionally severe SCD sequelae such as of stroke [7] and neurocognitive compromise [8].

SCA is genetically characterised by a single point mutation in the globin locus, however, there are additional genetic factors that modulate the clinical course of this disease and patients manifest widely varying degrees of severity. Several genomic loci have now been identified that are associated with increased levels of fetal haemoglobin (HbF) and as a consequence influence the clinical course of SCA [9]–[11]. HbF is a highly heritable trait; based on samples of Europeans with a normal hematocrit 89% of the variation is genetically determined [12]. To date at least 3 quantitative trait loci (QTL) have been shown to be associated with persistence of elevated HbF and account for 20–50% of the inter-individual variation in HbF. These loci include the combined *XmnI-158* and *OR51B5/6* locus on chromosome 11p15.4 [13], the *HBS1L-MYB* intergenic polymorphism (*HMIP*) locus on chromosome 6q23.3 [14], which may reflect the role of a single variant, and the *BCL11A* locus on chromosome 2q16.1 [15], [16]. In order to stimulate HbF expression, experimental and clinical work resulted in adoption of hydroxyurea as adjunct treatment for high risk SCA patients [17], and this is now widely accepted therapy. Moreover, other investigators have recently demonstrated that inactivation of *BCL11A* in an SCD transgenic mouse model completely eliminates the hematologic and pathologic defects associated with SCD through induction of high-level pancellular HbF [18].

SCA was the first molecular disease to be recognized in medical practice and the description of the DNA-protein relationship underlying this disorder was a milestone in molecular medicine. It is both ironic and unfortunate, therefore, that the powerful new tools of genomics have not been applied to research on SCA on the African continent. To the best of our knowledge, only one study has reported data on selected genetic effects associated with increased level of HbF amongst SCA patients in sub-Saharan Africa [19] and no studies on the association of these SNPs with clinical outcomes has yet been reported from the region. The sickle mutation exists in Africa on diverse genetic haplotype backgrounds [20] and geographically separate African populations may vary in the effects of linked genetic variants found on specific haplotypes. Studies of SCA in African sub-populations could therefore potentially lead to important new findings about factors that influence the disease phenotype. The aim of this study was to explore the frequency and influence of 10 SNPs known to influence HbF within the *HMIP, BCL11A, XmnI-158* and *OR51B5/6* loci amongst a group of Cameroonian SCA patients and to compare these data to a well described cohort of African-American patients in the Cooperative Study of Sickle Cell Disease (CSSCD).

## Materials and Methods

### Ethics Statement

The study was performed in accordance with the guidelines of the Helsinki Declaration. Ethical approval was given by the National Ethical Committee Ministry of Public Health, Republic of Cameroon (No 033/CNE/DNM/07); and the University of Cape Town, Faculty of Health Sciences Human Research Ethics Committee (HREC RE: 132/2010). Written and signed informed consent was obtained from the adult participants who were 18 years or older, and for the children consent was obtained from parents/guardians with an assent from the participants older than seven years of age.

### Patients and assessment of clinical events

The study was conducted at the Yaoundé Central Hospital and Laquintinie Hospital in Douala, the primary port and second largest city in Cameroon. Socio-demographic and clinical data were collected by means of a structured questionnaire. Parents/guardians as well as adult SCA patients were interviewed; patients’ medical records were reviewed, to delineate their clinical features over the past three years. Specifically, the occurrences of vaso-occlusive painful crisis (VOC), hospital outpatient visits, hospitalisations, overt strokes, blood transfusions and administration of hydroxyurea were recorded. VOC events were defined as episodes that could not be attributed to causes other than SCA and required a hospital visit and treatment with prescription analgesics. Anthropomorphic variables (body mass Index (BMI), and blood pressures (BP) were measured in the outpatient setting.

The sampling strategy was not restricted to hospital-based patients in an attempt to avoid the bias that might result from including only the sickest patients. To accomplish this goal two SCA patients’ associations in Cameroon were engaged for collaboration and additional patients were recruited during their monthly meetings. No incentive was provided for participation in the study. Only patients who had not received a blood transfusion or hospitalisation in the past 6 weeks where included. None were currently treated with hydroxyurea.

### Hematological Phenotypes

Hemoglobin electrophoresis and complete routine blood count of the SCA patients were conducted upon arrival at the hospital. Two methods of HbF detection were employed in this study for successive groups of patients; initially, as a result of limited capacity, the alkali denaturation test (ADT) was used, and subsequently high performance liquid chromatography (HPLC) was performed. HbF determinations were performed at the hematological laboratory of the Centre Pasteur in Yaoundé; we excluded from the analysis measurements done in patients <5 years old because HbF levels are not yet stable at this age. HbF levels were measured by ADT in 344 patients (55.5%). HbF measurements by HPLC and ADT techniques were found to have differences in median values (P = 0.001), with ADT yielding the median value of 11.7% compared to 6.8% for the HPLC method.

As previously reported, HbF was measured in the CSSCD samples by ADT and HbA2 was measured by Diethylamino Ethanol column chromatography [21]. For HbF in the CSSCD study, we also excluded from the analysis measurements done in patients <5 years old [9].

### Genotypes

#### Cameroonian patients

DNA samples were extracted from peripheral blood, following instructions for the commercial kit (Puregene blood kit (Qiagen, USA) at the molecular diagnostic laboratory, Gyneco-Obstetric and Paediatric Hospital, Yaoundé, Cameroon. Genotype analyses were performed in the Division of Human Genetics, Faculty of Health Sciences, University of Cape Town.

#### Molecular Diagnostic Testing for SCA (HbSS)

Analysis for presence of the sickle mutation was carried out on 200 ng DNA by PCR, on a thermocycler (BIORAD, USA) to amplify a 770 bp segment of the β-globin gene, followed by Dde I (GIBCO-BRL, USA) restriction analysis of the PCR product. Only patients SCD-HbSS were included in the analysis, according to a previously reported method [22].

#### Haplotyping of the β-globin gene cluster

Five restriction fragment length polymorphism (RFLP) regions in the β-globin gene cluster were amplified using published primers and methods to analyse the XmnI (5'Gγ), HindIII (Gγ), HindIII (Aγ), HincII (3˙'Ψβ), and HinfI (5'β) [23]. RFLP sites and the fragments were visualised by agarose gel electrophoresis; β-globin gene haplotypes were defined by examining the combination of the restriction sites.

SNPs genotyping in the HMIP, BCL11A, XmnI-158 and OR51B5/6 loci: For the purpose of obtaining genotype for variants associated with HbF levels that had been previously identified in SCA patients, ten regions containing specific SNPs were amplified: viz, for the *BCL11A* locus, SNPs rs11886868 and rs4671393; for the *HMIP1/2* loci: SNPs rs28384513, rs9376090, rs9399137, rs9389269; rs9402686 and rs9494142; for the *OR51B5/6* loci: SNP rs5006884, for *HBG* loci, SNP rs7482144.

PCR was performed to determine genotypes using SNaPshot multiplex ready reaction mix (Applied Biosystems, California, USA) as previously reported [24]. For SNP genotyping, the accuracy of the SNaPshot method was shown to be comparable to Biplex Invader and Pyrosequencing, while lower in cost when multiplex reactions are used [24]. Two singleplex reactions allowed the amplification of SNPs rs7482144 and rs9399137; and three multiplex reactions allowed the amplification of rs11868668, rs28384513 and rs9389269 (mutiplex1); rs9376090, rs9402686 and rs5006884 (mutiplex 2); rs9494142 and rs4671393 (mutiplex 3).

Capillary electrophoresis was used for the determination of genotype; 0.5 μl treated SNaPshot reaction mixture was mixed with 5 μl HiDi formamide (Applied Biosystems) and 0.2 μl GeneScan -120 Liz size standard. This mixture was loaded onto a Micro Amp 96 well reaction Plate (Applied Biosystems) and analysed using the GeneScan-120 Liz and run model at E5_36_POP7.

#### Cycle sequencing

For Cameroonian samples, we confirmed the genotyping results for both SNaPshot and RFLP-PCR by sequencing a sub-set of samples (10%). Sequencing was performed using BigDye terminator mix (Promega, Wisconsin, USA) and 5X BigDye terminating buffer (Promega, Wisconsin, USA). Cycle sequencing was then performed on the Applied Biosystems thermal cycler (Gene Amp PCR system 9700). An electropherogram was generated using sequence analysis software and imported into Bioedit Sequence alignment editor 7.0.0 (Tom Hall, Isis Pharmaceuticals, Inc.) for the alignment of the sequence with a reference sequence to identify the SNP of interest.

#### CSSCD patients

For the CSSCD patients, all DNA genotyping was performed by using the mass spectrometry-based MassArray iPLEX platform from Sequenom as previously reported [9]. For SNPs passing quality control, the genotyping success rate was >93% and the consensus error rate, estimated from replicates, was <0.3%.

### Statistical analysis

Descriptive statistics were obtained for all quantitative data using SPSS (IBM, USA version 21.0). Normality was confirmed by the Shapiro-Wilk Test followed, by the use of parametric (Chi-squared test and t-test) or non-parametric tests (Mann-Whitney U test for 2 samples or the Kruskal-Wallis ONE way ANOVA for more than 2 samples). Significance was set at the 0.05 level. Genetic analysis was performed with PLINK software [25], testing only the additive genetic model under a linear regression framework. For quality control, a Hardy-Weinberg test was performed on all genotype results: two SNPs were dropped because of significant violation of HWE (rs1188686 in BCL11A, MAF 0.31; HWE P-value: 0.000609; and rs9389269 in the *HBS1L-MYB* locus, MAF: 0.17; HWE P-value: 2.42E-05). A third SNP was monomorphic (rs9376090 in the *HBS1L-MYB* locus, all the patients were T/T homozygous). In the CSSCD patients, on Hardy-Weinberg equilibrium test, *P* value was >0.05 for all SNPs, except for the -158 (G>A) *Xmn*I polymorphism in the *G*γ-*globin* (*HBG2*) gene promoter (rs7482144), which was in disequilibrium (*P*  =  0.002) [9]. For a polymorphism in LD (linkage disequilibrium) with the sickle cell mutation, this finding is expected in a population of SCD patients [9].

When several SNPs were genotyped at the same locus, we used conditional analysis and stepwise regression to determine whether one or more independent association signals were present.

To correct for the skewness of the HbF distribution, we log10-transformed and normalized the data to obtain the quantitative trait used in the association analysis (after correcting for age, gender, and electrophoresis technique and history of transfusion). Statistical models to investigate the relationship between HbF-associated SNPs and SCD complications were conducted in the statistical package R, version 2.5.1.

## Results

### Socio-demographic and Hematological variables

The description of the study sample is presented in Table 1. Among the 610 patients 50.3% were female; the mean age (± SD) of patients was 17.3±10 years (range: 5–54 years), and the majority of patients were children aged 5–10 years (32.2%; *n* = 196) or adolescents 11–20 years (50%; *n* = 305). Patients lived mostly in the urban areas of Yaoundé and Douala (93%; *n* = 567), the two largest cities in Cameroon. Among the participants, 41% (*n* = 238), 45.6% (*n* = 265) and 13.2% (*n* = 78) attended formal primary, secondary or tertiary education levels, respectively.

**Table 1:** Cohorts’ description.

Variables	Cameroon		CSSCD	
	N	Mean±SD	N	Mean±SD
M/F	303/307		682/593	
Age (Yrs)	610	17.3±10	1275	14.5±12.1
RBC (10^12^/L)	610	2.8±0.7	1275	2.8±0.6
Hb (g/dl)	610	7.8±1.6	1275	6.4±4.7
MCV (fl)	610	84.4±9.9	1275	89.4±9.0
MCHC (g/dl)	610	34.0±3.8	1275	30.1±2.9
WBC (10^9^/l)	610	13.7±5.6	1275	11.9±2.6
Monocytes (10^9^/l)	610	1.5±1	1275	0.7±0.5
Pl atelets (10^9^/l)	610	374.6±123	1275	442±151
HbA2 (%) HPLC*	244	3.3±1.3	1275	2.9±0.6
HbF (%) (HPLC)*	244	7.5±4.8	1275	6.3±4.6
N VOC/Yr	572	3±3	1275	0.7±1.4
N hospital attendance (per Yr)	608	2.2±3.2		NA
N hospital admission (per Yr)	606	2.3±3		NA
N patients with Strokes	25/608	4.1%	46/1229	3.6%

*Hb electrophoresis was also obtained from 344 patients (55.5%) using alkali denaturation test (ADT), with a mean of 11.4±9.4 for HbF and 4.1±2.1 for HbA2 levels. For the analysis, to correct for the skewness of the HbF distribution, we log10-transformed and normalized the data to obtain (after correcting for age, gender, and electrophoresis technique and history of transfusion) the quantitative trait used in the association analysis. NA =  Not Applicable.

Marital status of parents was distributed as follows: married (58%; *n* = 235), single (28%; *n* = 113), widows (9%, *n* = 36) and divorced (5%; *n* = 21). Forty two percent (*n* = 158) of fathers and 23% (*n* = 108) of mothers were formally employed, while 41% (*n* = 153) of fathers and 39% (*n* = 183) of mothers were working in the informal sector. The majority of parents (75%) earned monthly direct incomes that were < 300 USD. The median age at diagnosis of SCA, based on haemoglobin electrophoresis, was 3 years (range: 1month - 29 years). Only 31% (*n* = 180) of patients were diagnosed before their first year of life.

After molecular analysis, the vast majority of patients were determined to have SCA (HbSS) (ie, 97.4%; *n* = 594); 15 (2.4%) patients were HbS-β thalassemia and one patient had an HbSC genotype.

There were no significant differences among the socio-demographic variables (marital status of parents, employment status, <300 USD direct revenue; urban vs. rural), in association with HbF levels or in association with the clinical events (number of VOC, number of hospital attendance or number of hospitalisations, overt strokes). The hematological parameters were generally comparable to those of African Americans in the CSSCD; the WBC count and the occurrence of VOC were somewhat more frequent in the Cameroonians (Table 1).

### Haplotypes in the beta-globin gene cluster

Based on analysis of 1082 chromosomes, the allele frequencies of various haplotypes in the beta-globin genes cluster showed that Benin (74%; *n* = 799) and Cameroon (19%; *n* = 207) were most prevalent forms, followed by atypical haplotype (6%; *n* = 66). Bantu (*n* = 5); Senegal (*n* = 3) and Indian-Arab (*n* = 2) alleles were very rare.

In combination, the Benin/Benin haplotypes represented 57% (*n* = 307), Benin/Cameroon 25% (*n* = 137), Benin/atypical 8% (*n* = 45) and Cameroon/Cameroon 5% (*n* = 26) haplotypes were most prevalent. There were no significant differences in the association with clinical events or HbF levels among the haplotype combinations.

### Association of Genetic Variants to HbF Levels

**Figure 1:**
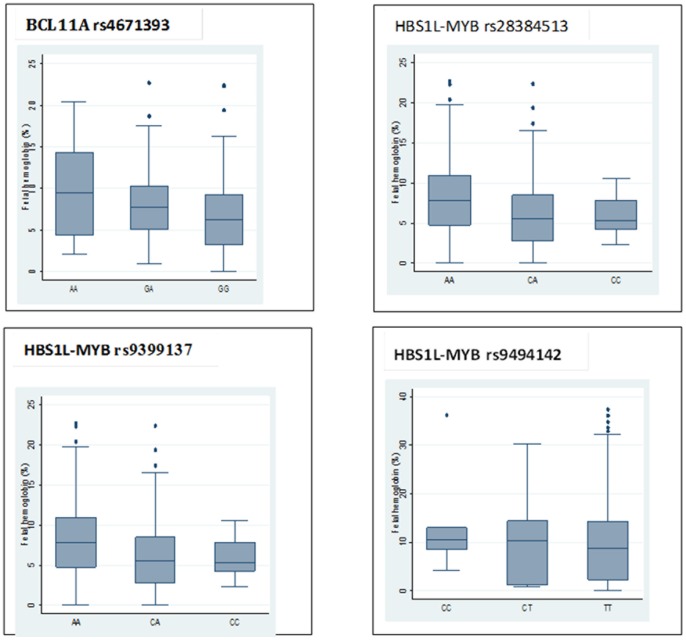
Distribution of fetal hemoglobin levels conditioned on SNP genotypes. Boxes have lines at the lower quartile, median, and upper quartile. The plot whiskers extend up and down from the median a distance 1.5 times the interquartile range of the boxes (truncated at zero where necessary). Outliers are the points outside the whiskers indicated as circles.

We did not replicate the association of rs7482144 in *HBG2* to the HbF levels. The adjacent rs5006884 *OR51B5/6*, was also not significantly associated to HbF level in Cameroonian SCA patients (Table 2). On the other hand, the two principal known HbF loci were significantly associated in the study patients (Table 2). The effect of minor alleles at this these loci resulted either in a depression of mean HbF values (rs28384513, *HBS1L-MYB*), or an increase in HbF (rs4671393 in *BCL11A;* rs9399137 and rs9494142 in *HBS1L-MYB*) (figure 1). As previously reported in African Americans at the *BCL11A* loci, when we conditioned the association analysis on rs4671393, rs11886868 was no longer significant, suggesting that these markers tag the same causal polymorphism n this Cameroonian SCA cohort.

**Table 2:** Fetal hemoglobin association results for SNPs at the BCL11A, HBS1L-MYB, and beta-globin loci in the CSSCD and the Cameroon sickle cell Anaemia cohort.

SNPs					HbSS Cameroon (N = 596)			HbSS CSSCD (N = 1275)			
Locus	SNP	Position&	Allele Change	MAF$	Effet Size#(SE)	Variance Explamined (%)	P values	MAF	Effet Size (SE)	Variance Explamined (%)	P values
**Chr. 2** &											
***BCL11A***	rs11886868+	60720246	T>C	0.31	0.167 (0.06685)	1.4	0.01	0,31	0.524 (0.041	11.8	4.00E-35
***BCL11A***	**rs4671393**	**60720951**	**G>A**	**0.30**	**0.201 (0.07322)**	**1.7**	**0.01**	**0.27**	**0.598 (0.042)**	**14.1**	**2.00E-42**
**Chr. 6**											
***HBS1L-MYB***	**rs28384513**	**135376209**	**A>C**	**0.2**	–**0.3002 (0.08189)**	**3**	**0**	**0.2**	–**0.102 (0.049)**	**0.4**	**0.04**
***HBS1L-MYB***	rs9376090	135411228	T>C	0		NA		NA	NA	NA	
***HBS1L-MYB***	**rs9399137**	**135419018**	**T > C**	**0.043**	**0.412 (0.1562)**	**1.6**	**0.01**	**0.06**	**0.571 (0.086)**	**3.5**	**5.00E-11**
***HBS1L-MYB***	rs9389269+	135427159	T>C	0.18	0.09561 (0.08244)	0.3	0.25	ND	ND	ND	ND
***HBS1L-MYB***	rs9402686	135427817	G>A	0.03	0.1447 (0.1887)	0.1	0.44	ND	ND	ND	ND
***HBS1L-MYB***	**rs9494142**	**135431640**	**T>C**	**0.11**	**0.3391 (0.111)**	**2.1**	**0**	**ND**	**ND**	**ND**	**ND**
**Chr.11**											
***HBG2***	rs7482144	5276169	G>A	0.005	–0.05843 (0.5031)	0	0.91	0.07	0.407 (0.080)	2.2	4.00E-07
***OR51B5/6***	rs5006884	5373251	C>T	0.08	0.04163 (0.1246)	0	0.74	ND	ND	ND	ND

NA =  Not applicable. Monomorphic T for the entire sample.

ND  =  Not determined.

& Chr.  =  chromosome; Position on NCBI Build 36.1.

$ MAF, minor allele frequency. Minor alleles (positive strand) are given in the parentheses.

# Effect sizes and standard errors are given in standard deviation units for the minor allele.

+ On QC, these two SPNs were out of HWE.

The largest allelic effect (0.41, Table 2) in the Cameroonian patients was detected at the *HBS1L-MYB* locus, rs9399137; leading to changes in median values of HbF of 10.6%, 10.3% and 8.7% for the C/C, C/T and TT alleles, respectively. We further explored whether the multiple variants at the *HBS1L-MYB* locus represented independent signals of association. Using stepwise regression, we found that rs28384513, rs9399137, rs9376090, rs9389269 rs9402686 and rs9494142 are independent association signals in the Cameroonian sample (Table S1 and Figure S1).

We disaggregated the patient sample, based on the HbF assessment technique (ADT vs HPLC), and found that the significant associations with HF levels, examined independently, were present in both sub-groups studied using the different assay methods, in rs11886868 (*BCL11A*), rs4671393 (*BCL11A),* rs28384513 (*HMIP 1*) *and* rs9494142 (*HMIP 2*) (Table S2).

### HbF-associated variants and hematological parameters

We tested correlations between the 10 HbF-associated SNPs and various blood cell parameters [RBC count, WBC count, mean corpuscular volume (MCV), mean corpuscular hemoglobin (MCH), platelet count, and monocyte levels]. *BCL11A* rs4671393 was associated with a wide range of haematological indices (Table 3). In addition, rs9402686 (*HMIP 2*) was significantly associated with platelet count; rs28384513 (*HMIP 1*) with WBC counts, and rs9399137 (*HMIP 2*) with Hb levels, rs9402686 (*HMIP 2*) and rs9494142 (*HMIP 2*) were both associated with MCV.

**Table 3:** BCL11A (rs4671393) affected a wide range of haematological variables.

GENOTYPES	Hemotological variables									
	RBC (10^12^/l)		Hemoglobin (g/dl)		Leucocytes (10^9^/l)		Lymphocytes (10^9^/l)		Platelets (10^9^/l)	
rs4671393 (BCL11A)	Median	P	Median	P	Median	P		P	Median	P
CC	2.9	0.02	8.1	0.02	13.1	0.01	6.1		316	
CT	2.7		7.8		12.6		5.1	0.02	393	0.02
TT	2.6		7.5		13.6		5.5		385.5	

### Effect of HbF-associated variants on pain crises and rate of hospitalization

**Figure 2:**
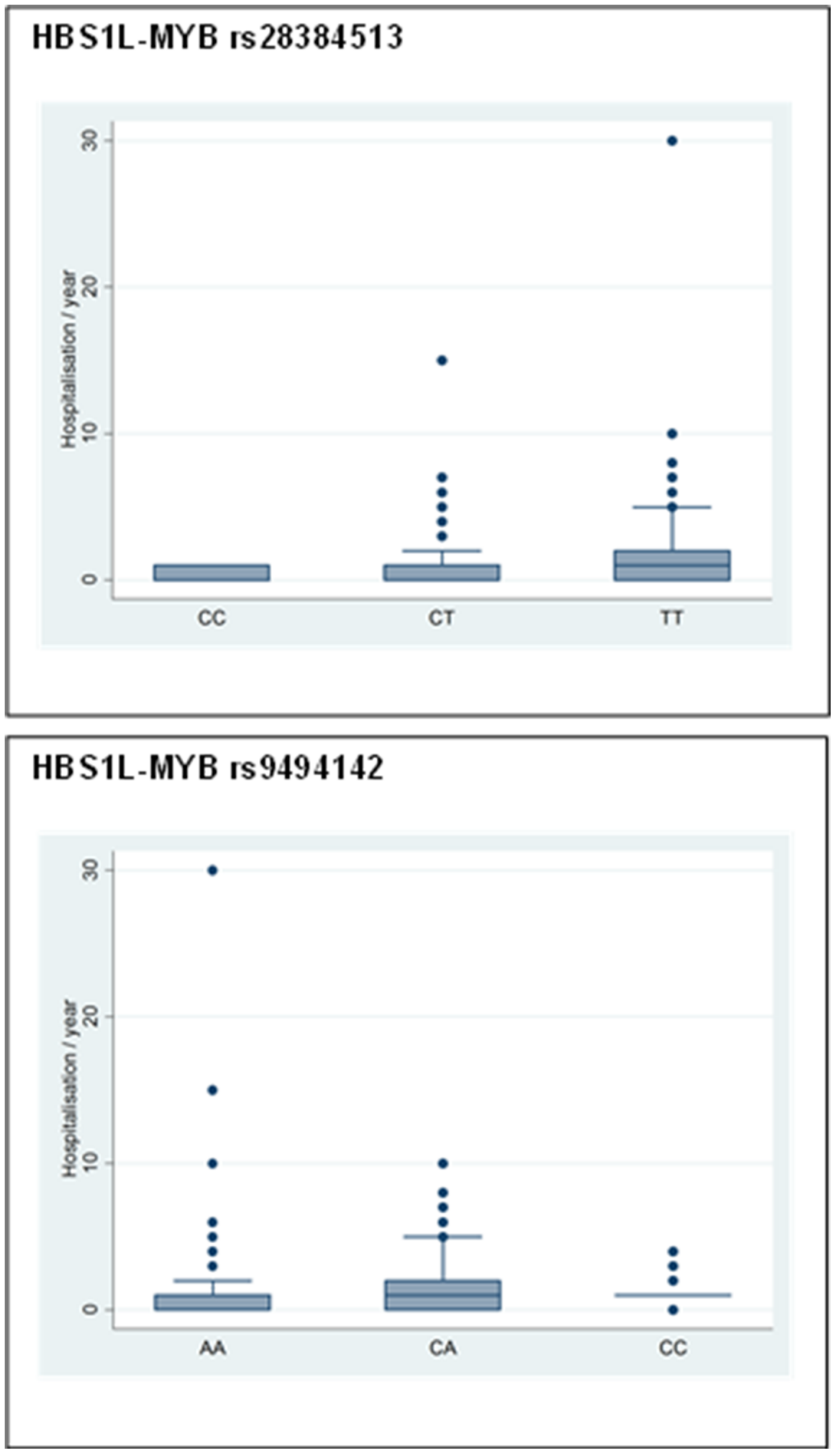
Association of SNPs in *HBS1L-MYB* locus and Rates of Hospitalization. Two specific SNPs were associated, with rate of hospitalization, a potential marker of overall disease severity. Boxes have lines at the lower quartile, median, and upper quartile. Outliers are the points outside the whiskers indicated as circles.

Two SNPs [rs28384513 (*HMIP 1*) and rs9494142 (*HMIP 2*)] were associated with the number of hospitalisations (P = 0.028 and P = 0.04, respectively, independent of HbF level and various socio-demographical parameters (figure 2; Table S3). We did not, however, find an association between the genotyped SNPs and the numbers of VOC, and overt stroke episodes. In addition, there was no differential in MAF in the three loci across various age ranges suggesting that there is not a strong selective advantage for these loci.

## Discussion

Considerable progress has been made in understanding disease modifying genetic factors in SCD, prompting speculation that this information may be of potential therapeutic value. In an earlier study, Thein and colleagues concluded that at least three independent genetic variants (rs28384513, rs9399137 and rs6929404) explain the linkage peak observed at 6q23 in the *HBS1L-MYB* intergenic region associated with HbF levels [14]. An association signal with HbF levels in the *HBS1L-MYB* intergenic region was also identified in a large non-anemic Sardinian cohort [26]. Lettre *et al.* [9] genotyped SNPs rs28384513 and rs9399137, originally reported by Thein et al. [14] as well as three SNPs reported in the Sardinian study (rs7776054, rs9389268, and rs4895441), in the US CSSCD and a Brazilian SCD cohort and replicated the association with HbF levels [9]. Moreover, Lettre *et al*. also explored the intronic SNP in *BCL11A*, rs11886868, that strongly correlates with HbF levels in the CSSCD and confirmed that rs4671393 in *BCL11A* provides a strong signal of association with HbF levels in SCD populations [9]. In a Tanzanian SCD cohort, SNPs in *HBS1L-MYB* and *BCL11A* were also found to have a significant impact on the HbF level [19]. Most recently, researchers found that common genetic variation at *BCL11A* associated with HbF levels lies in noncoding sequences associated with an erythroid enhancer chromatin signature, this led to the suggestion that the GWAS-marked *BCL11A* enhancer represents an attractive target for therapeutic genome engineering for the β-hemoglobinopathies [16].

In the present study, the first to our knowledge examining these genetic relationships in West Africans, we examined the effect of 10 common genetic polymorphisms on HbF and various clinical outcomes in SCA patients from Cameroon. We have confirmed robust genetic associations in *BCL11A* and *HBS1L-MYB* intergenic region loci, as previously reported in African Americans and Afro- Brazilians [9] and Tanzanians [19]. In the CSSCD, five SNPs explained >20% of residual HbF variation; in the present study 4 SNPs explained about 10% of HbF variation. As a correlate, in the present study, we found much lower effect sizes for the relevant markers, especially in the *BCL11A* loci (Table 2); this could in part be due to our smaller sample size, or to the reduction in precision through the use of ADT to measure HbF levels in the majority of patients [27]. Nevertheless, HbF was measured in the CSSCD samples in the 1980s by ADT [21]; thus, the difference in effect size could well be a valid finding that deserves further investigation; additional fine-mapping of *BCL11* should therefore be considered in future studies. Alternatively, another potential explanation could be possible poor assay precision of ADT techniques to measure HbF on the Cameroonian samples. Despite this possible limitation in HbF phenotyping, the replication of this level of genetic effect sizes for human complex quantitative traits, explained using a few genetic polymorphisms, is relatively strong in comparison to many other quantitative human traits.

As also reported in the present study, multiple variants at the *HBS1L-MYB* locus were shown to be independent in African-American, Afro-Brazilian [9] and Tanzanian SCD patients [19]. Together the polymorphisms explained 8.3% of the variation in HbF levels in our SCA patients (Table 2). In addition, we have shown that rs9399137, which acts as a tagging SNP for the *HMIP-2* sub-locus in European populations [14], [26], occurred at a low frequency in this Cameroonian cohort, as noted previously in both African-American [9] and Tanzanian patients [19]; thus, rs9399137, is not an appropriate proxy for the causative sequence variant that influences HbF at *HMIP-2* on African chromosomes [28]. However, we report, in the *HMIP-2* sub-locus, a much higher frequency of rs9389269 (0.18) in Cameroonian as compared to the Tanzanian SCD patients (0.03); this observation could indicate a high degree of variation in the MAF of this SNP amongst SCD patients from various African population groups. Specifically, a 3 bp deletion in perfect LD with rs9399137 in Chineses, Europeans and some Africans was reported to be the candidate functional motif for the *HMIP-2* region [29]. But, in the current Cameroonian samples, rs9389269 was not linked to rs9399137 (r^2^  = 0; D’ = 0.138; Table S1). Using 1000Genome data (Phase I Release Version 3 20101123), we also estimated the LD between rs9389269 and rs9399137 among Luhya in Webuye from Kenya (AWK) and Yoruba in Ibadan from Nigeria (YRI); equally, the results did not indicate any linkage between the two SNPs in any of the two native African populations: r2 = 0.012; D' = 0.233 for AWK and r2 =  0.002; D' = 1.000 for YRI. Thus, the candidate for functional motif for the *HMIP-2* region in sub-Saharan African populations deserves to be explored in future study.

The importance of the *HMIP* locus in African populations might therefore have been underestimated by datasets using markers tailored to European studies [19]. Fine-mapping this locus could lead to new findings in African patients with SCD.

The association between the XmnI polymorphism (rs7482144) in the proximal promoter of the γ-globin (*HBG2*) gene and HbF levels is well documented in SCA patients [9], [20]. Due to the virtual absence of the Senegal and Indian Arab haplotypes that contain the XmnI variant, we lacked power to replicate the association of rs7482144 in *HBG2* with HbF levels; in the CSSCD, rs7482144 explains 2.2% of the variation in HbF levels. Other African populations with the Senegal S haplotype that contains the rs7482144 SNP would be better suited to study the effects of this variant. Similarly, a strong signal adjacent to the *HBB* cluster, recently detected in African-American patients at rs5006884 in *OR51B5/6* [13], was not significant in our patients. In a report that included Tanzanian patients, the positive association of HbF with rs5006884 disappeared when linkage disequilibrium with rs7482144 was taken into account [19]; this additional HbF locus does not seem to play an important role in Cameroonians or Tanzanians.

We also described the prevalence of various haplotypes in the β-globin gene cluster in Cameroonian SCD patients and reveal that the Benin haplotype was the most prevalent. It is perhaps anomalous that the “Cameroon” haplotype, which seems to occur more frequently in Sudan [30], is not dominant in this population. The results raise the question of the geographical origin of this haplotype and also suggest that the detailed geographic distribution of known sickle haplotypes is still not well established. The refinement of the molecular structure (i.e. haplotype) in *HBB* and migration patterns may yield important information about the evolutionary history of SCA that could also be clinically relevant [31]. Contrary to our findings, a recent report argues that the S haplotype itself (beyond HbF regulation) correlates with severity [32]. Differences in haplotypes and SNP allele frequencies between SCA patients from different geographic populations underscore the need to conduct GWAs in regional sub-Saharan African populations.

Understanding the effect that HbF-associated variants have more generally on blood cell production and other parameters will be important in our efforts to define their biological roles and for the development of targeted therapies [9],[16]. As in African Americans and Afro-Brazilians studies [9], we replicated the finding that in combination *HBS1L-MYB* rs9399137, and *BCL11A* rs4671393 SNPs affected platelet counts. In addition *BCL11A* rs4671393 SNPs was associated with wider range of hematological indices in Cameroonians (Table 3), independently of HbF level. Significant associations of *HBS1L-MYB* rs9399137 with RBC indices (RBC count, MCV, MCH), and platelet and monocyte counts have been reported in healthy non-anemic Europeans [14]. However, in African-American and Brazilian patients Lettre et al. did not replicate these findings [9].

Previous analyses of the CSSCD dataset showed that increased HbF levels correlate with less severe complications, fewer pain crises [33] and improved survival [34]. Having shown that four sequence variants at two loci influence HbF levels (table 2; Fig. 1), we next asked whether these SNPs correlate with pain crisis or stroke in SCA: we did not find any significant associations. However, two SNPs in *HBS1L-MYB* were associated with the number of hospitalisations (figure 2); if confirmed, this could be the first evidence of the clinical effect that is associated with these variants. This result could suggest that clinical genotyping of these variants, and others, yet to be found, may potentially be useful to risk stratify SCD patients, and to serve as a guide to adjusting therapeutic and follow up strategies. This finding deserves further investigation with a larger sample or, ideally, a prospective birth cohort, to fully appreciate the potential clinical value. Clearly much remains to be learned about the myriad genetic factors which modify the course of SCA in Africans.

### Limitations and perspectives

A limitation of this study was our inability to use the HPLC method to measure HbF in all patients; this method was not available in Cameroon at the beginning of the sample collection. Even though we have shown that, use of the older assay did not affect the main outcomes ie, the association of targeted SNPs to HbF (supplementary data 2). The systematic use of HPLC for HbF measurement should have allowed more precise estimation of effect sizes of the genotyped SNPs as well as appropriate comparison to the other populations of SCA patients. In addition, phenotyping HbF levels with ADT instead of HPLC in the majority of samples could have affected the study of the associations of haplotypes, HbF levels and various clinical events. Self-report of clinical variables like VOC episodes can also lack precision and pain tolerance and financial factors could have been limiting factors for hospital attendance. However, it has also proven difficult to detect an association of genetic markers with VOC in affluent settings [9] [35].

Despite the above limitations, this study represent an important step forward in the understanding of influence of SNPs in modifying genes on HbF level in Cameroon, and in sub-Saharan African patients generally. While more than 70% of SCA patients world-wide live in Africa, most advances in the molecular understanding and management of SCA have been based on research conducted in either the US or the UK. Clearly contemporary research tools must now be widely implemented in Africa. This study therefore also has a capacity-building dimension, as it was fully performed from design, molecular analysis and reporting, on the African continent. Increased capacity to conduct both clinical and laboratory research on SCA in Africa could in turn create major collaborative research opportunities at the regional and international levels.

## Conclusion

This study has confirmed the associations of SNPs in *BCL11A* and *HBS1L-MYB* and fetal haemoglobin in Cameroonian SCA patients and the association of two specific SNPs with rate of hospitalization, a potential marker of overall disease severity. These results highlight the differential frequency of SNPs associated to HbF levels amongst various demographic groups of SCA patients. Additional data in other African site are needed to define an updated map of these variants on the continent, as well as potential new loci that could influence fetal hemoglobin.

## Supporting Information

Table S1 **Linkage analyses of the targeted of SNPs pairs, studied in the** ***BCL11A*** **and** ***HBS1L-MYB*** **loci.**
(DOCX)

Table S2 **HbF assessment technique and SNPs Associations.**
(DOCX)

Table S3 **Effect of HbF-associated variants on pain crises and hospitalization rates.**
(DOCX)

Figure S1 **Analysis showed that rs28384513, rs9399137, rs9376090, rs9389269 rs9402686 and rs9494142** ***HBS1L-MYB*** **loci are independent association signals the studied sample.**
(DOCX)
