# Supplementary material for: Association of Variants at BCL11A and HBS1L-MYB with Hemoglobin F and Hospitalization Rates among Sickle Cell Patients in Cameroon
Source: PLoS One. 2014 Mar 25;9(3):e92506. doi: 10.1371/journal.pone.0092506 (PMC3965431; doi:10.1371/journal.pone.0092506)
Supplement: Table S1 — Linkage analyses of the targeted of SNPs pairs, studied in the BCL11A and HBS1L-MYB loci. (DOCX) [file pone.0092506.s001.docx]

Table S1 : linkage analyses of the targeted of SNPs pairs, studied in the *BCL11A* and *HBS1L-MYB* loci

| **Paire de SNP** | **Coefficients de DL** |
| --- | --- |
|  |  |
| **rs4671393-rs11886868** |  |
| **D'** | 0.928 |
| **r2** | 0.815 |
| **rs28384513-rs9376090** |  |
| **D'** | 0.978 |
| **r2** | 0.003 |
| **rs28384513-rs9399137** |  |
| **D'** | 0.474 |
| **r2** | 0.003 |
| **rs28384513-rs9389269** |  |
| **D'** | 0.404 |
| **r2** | 0.009 |
| **rs28384513-rs9402686** |  |
| **D'** | 0.994 |
| **r2** | 0.009 |
| **rs28384513-rs9494142** |  |
| **D'** | 0.718 |
| **r2** | 0.018 |
| **rs9376090-rs9399137** |  |
| **D'** | 1.000 |
| **r2** | 0.022 |
| **rs9376090-rs9389269** |  |
| **D'** | 0.998 |
| **r2** | 0.004 |
| **rs9376090-rs9402686** |  |
| **D'** | 1.000 |
| **r2** | 0.027 |
| **rs9376090-rs9494142** |  |
| **D'** | 1.000 |
| **r2** | 0.007 |
| **rs9399137-rs9389269** |  |
| **D'** | 0.138 |
| **r2** | 0 |
| **rs9399137-rs9402686** |  |
| **D'** | 0.069 |
| **r2** | 0.004 |
| **rs9399137-rs9494142** |  |
| **D'** | 0.068 |
| **r2** | 0.002 |
| **rs9389269-rs9402686** |  |
| **D'** | 0.929 |
| **r2** | 0.14 |
| **rs9389269-rs9494142** |  |
| **D'** | 0.229 |
| **r2** | 0.033 |
| **rs9402686-9494142** |  |
| **D'** | 0.902 |
| **r2** | 0.21 |
