# Supplementary material for: Association of Variants at BCL11A and HBS1L-MYB with Hemoglobin F and Hospitalization Rates among Sickle Cell Patients in Cameroon
Source: PLoS One. 2014 Mar 25;9(3):e92506. doi: 10.1371/journal.pone.0092506 (PMC3965431; doi:10.1371/journal.pone.0092506)
Supplement: Table S2 — HbF assessment technique and SNPs Associations. (DOCX) [file pone.0092506.s002.docx]

| **GENOTYPES** | **HbF ADT (%)** | | | **HbF HPLC (%)** | | |
| --- | --- | --- | --- | --- | --- | --- |
|  | **N** | **Median** | **P values** | **N** | **Median** | **P values** |
| **rs11886868 (BCL11A)** |  |  |  |  |  |  |
| **CC** | **36** | 15.5 | **0.0001** | **21** | 9.6 | **0.008** |
| **CT** | **117** | 13.6 |  | **79** | 7.7 |  |
| **TT** | **187** | 8.6 |  | **118** | 5,6 |  |
| **rs4671393(BCL11A)** |  |  |  |  |  |  |
| **AA** | **18** | 16.0 | **0,0001** | **19** | 9.5 | **0.02** |
| **GA** | **141** | 14.1 |  | **89** | 7.7 |  |
| **GG** | **184** | 8.1 |  | **114** | 6.3 |  |
| **rs28384513(HMIP 1)** |  |  |  |  |  |  |
| **AA** | **210** | 14.1 | **0.0016** | 140 | 7.8 | **0.01** |
| **CA** | **121** | 12.7 |  | 75 | 5.3 |  |
| **CC** | **13** | 8.1 |  | 8 | 5.5 |  |
| **rs9376090(HMIP 2)** |  |  |  |  |  |  |
| **CT** | **1** | 0.8 | 0.3 | 0 |  | NA |
| **TT** | **338** | 11.7 |  | 212 | 7 |  |
| **rs9399137 (HMIP 2)** |  |  |  |  |  |  |
| **CC** | 3 | 13 | 0.6 | 2 | 6,4 | 0.4 |
| **CT** | 25 | 11.7 |  | 13 | 6,7 |  |
| **TT** | 290 | 11.1 |  | 193 | 8.5 |  |
| **rs9389269 (HMIP2)** |  |  |  |  |  |  |
| **CC** | 20 | 12.0 | 1 | 16 | 7.8 | 0.9 |
| **CT** | 66 | 12.3 |  | 54 | 7 |  |
| **TT** | 245 | 11.1 |  | 140 | 6.8 |  |
| **rs9402686 (HMIP 2)** |  |  |  |  |  |  |
| **AA** | 0 | 14.1 | 0.2 | 1 | 7.3 | 1 |
| **GA** | 23 |  |  | 12 |  |  |
| **GG** | 321 | 11.1 |  | 205 | 6.8 |  |
| **rs9494142 (HMIP 2)** |  |  |  |  |  |  |
| **CC** | 3 | 12.5 | **0.02** | 2 | 13.9 | 0.4 |
| **CT** | 71 | 11.1 |  | 46 | 7.8 |  |
| **TT** | 267 | 8.0 |  | 166 | 6.8 |  |
| **rs7482144 (HBG 2)** |  |  |  |  |  |  |
| **GA** | 2 | 8.6 | 0.8 | 3 | 10.2 | 0.4 |
| **GG** | 315 | 11.7 |  | 193 | 7.2 |  |
| **rs5006884 (OR51B5/6)** |  |  |  |  |  |  |
| **CC** | 280 | 11.5 | 0.8 | 186 | 7.3 | 0.08 |
| **CT** | 61 | 11.9 |  | 33 | 5.2 |  |
| **TT** | 3 |  |  |  |  |  |

**Table S2. HbF assessment technique and SNPs Associations**
