# Supplementary material for: Association of Variants at BCL11A and HBS1L-MYB with Hemoglobin F and Hospitalization Rates among Sickle Cell Patients in Cameroon
Source: PLoS One. 2014 Mar 25;9(3):e92506. doi: 10.1371/journal.pone.0092506 (PMC3965431; doi:10.1371/journal.pone.0092506)
Supplement: Table S3 — Effect of HbF-associated variants on pain crises and hospitalization rates. (DOCX) [file pone.0092506.s003.docx]

| **GENOTYPES** | **Clinical parameters** | | | | |
| --- | --- | --- | --- | --- | --- |
|  | **N** | **Number of VOC per year** | | **Number of hospitalisation per year** | |
| **rs11886868 (BCL11A)** |  | **Median (range)** | **P values** | **Median(range)** | **P values** |
| **CC** | 56 | 2(0-9) | 0.58 | 1(0-6) | 0.65 |
| **CT** | 192 | 2(0-20) |  | 1(0-15) |  |
| **TT** | 300 | 2(0-30) |  | 1(0-30) |  |
| **rs4671393(BCL11A)** |  |  |  |  |  |
| **AA** | 36 | 2(0-40) | 0.53 | 1(0-10) | 0.81 |
| **GA** | 226 | 2(0-20) |  | 1(0-15) |  |
| **GG** | 293 | 2(0-30) |  | 1(0-30) |  |
| **rs28384513 (HMIP 1)** |  |  |  |  |  |
| **AA** | 344 | 2(0-40) | 0.64 | 1(0-30) | **0.028** |
| **CA** | 192 | 2(0-15) |  | 1(0-10) |  |
| **CC** | 21 | 2(1-30) |  | 1(0-4) |  |
| **rs9376090 (HMIP 2)** |  |  |  |  |  |
| **CT** | 1 | 2 | 0.89 | 0 | 0.25 |
| **TT** | 540 | 2(0-40) |  | 1(0-30) |  |
| **rs9399137 (HMIP 2)** |  |  |  |  |  |
| **CC** | 5 | 1(1-10) | 0.74 | 1(0-4) | 0.51 |
| **CT** | 35 | 2(0-10) |  | 1(0-10) |  |
| **TT** | 476 | 2(0-40) |  | 1(0-30) |  |
| **rs9389269 (HMIP2)** |  |  |  |  |  |
| **CC** | 36 | 2(0-20) | 0.59 | 1(0-6) | 1 |
| **CT** | 118 | 2(0-40) |  | 1(0-10) |  |
| **TT** | 377 | 2(0-30) |  | 1(0-30) |  |
| **rs9402686 (HMIP 2)** |  |  |  |  |  |
| **AA** | 1 | 2.5(0-12) | 0.35 | 1(0-5) | 0.91 |
| **GA** | 35 |  |  |  |  |
| **GG** | 516 | 2(0-40) |  | 1(0-30) |  |
| **rs9494142 (HMIP 2)** |  |  |  |  |  |
| **CC** | 5 | 2(0-4) | 0.66 | 0(0-1) | **0.04** |
| **CT** | 114 | 2(0-15) |  | 1(0-15) |  |
| **TT** | 426 | 2(0-30) |  | 1(0-30) |  |
| **rs7482144 (HBG 2)** |  |  |  |  |  |
| **GA** | 5 | 4(1-8) | 0.14 | 4(1-4) | **0.02** |
| **GG** | 500 | 2(0-40) |  | 1(0-30) |  |
| **rs5006884 (OR51B5/6)** |  |  |  |  |  |
| **CC** | 459 | 2(0-40) | 0.18 | 1(0-30) | 0.4 |
| **CT** | 91 | 2(0-15) |  | 1(0-15) |  |
| **TT** | 3 |  |  |  |  |

**Table S3. Effect of HbF-associated variants on pain crises and hospitalization rates**
