# Supplementary material for: Association of Variants at BCL11A and HBS1L-MYB with Hemoglobin F and Hospitalization Rates among Sickle Cell Patients in Cameroon
Source: PLoS One. 2014 Mar 25;9(3):e92506. doi: 10.1371/journal.pone.0092506 (PMC3965431; doi:10.1371/journal.pone.0092506)

**Figure S1. Analysis showed that rs28384513, rs9399137, rs9376090, rs9389269 rs9402686 and rs9494142 *HBS1L-MYB loci* are independent association signals the studied sample**


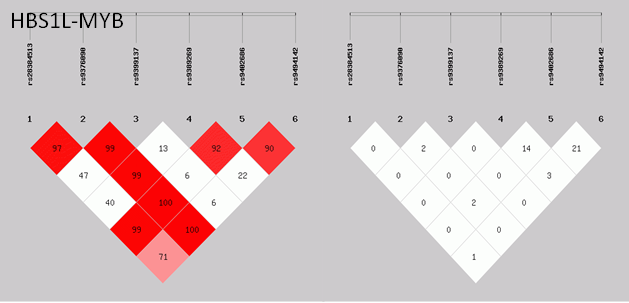

Supplement: Figure S1 — Analysis showed that rs28384513, rs9399137, rs9376090, rs9389269 rs9402686 and rs9494142 HBS1L-MYB loci are independent association signals the studied sample. (DOCX) [file pone.0092506.s004.docx]
